# Supporting a Student With School Absenteeism Through to Employment: A Case Report of Long-Term Home-Based Occupational Therapy for Emotion-Related School Attendance Challenges

**DOI:** 10.7759/cureus.114223

**Published:** 2026-08-09

**Authors:** Fugen Oto, Naoko Matsuda, Kaori Ito, Atsuko Morikawa, Hiromi Fujii

**Affiliations:** 1 Rehabilitation, Iroha Home-Visit Nursing Rehabilitation Station, Hiroshima, JPN; 2 Rehabilitation, K-Educational Foundation Morikawa High School, Hiroshima, JPN; 3 Rehabilitation, Kanon Co. Ltd, Hiroshima, JPN; 4 Occupational Therapy, Graduate School of Health Sciences, Yamagata Prefectural University of Health Sciences, Yamagata, JPN

**Keywords:** emotion-related school attendance challenges (er-sac), home-based occupational therapy, japanese rehabilitation, school absenteeism, vocational transition

## Abstract

In Japan, rising school absenteeism underscores the need for effective support systems. Students with neurodevelopmental disorders, such as autism spectrum disorder and attention-deficit/hyperactivity disorder, are highly vulnerable to prolonged absenteeism due to difficulties in interpersonal relationships and adaptation to their environment. However, existing literature primarily focuses on adaptation up to school reintegration or progression to higher education, with few studies examining the long-term transition from high school graduation to employment. This report describes the case of a 12-year-old boy with a neurodevelopmental disorder presenting with emotion-related school attendance challenges (ER-SAC). He received continuous home-based occupational therapy one to three times per week as part of a multidisciplinary support network and ultimately reached employment.

The intervention began by building rapport through his interests, subsequently addressing his daily life rhythm, activities of daily living, academic needs, and social participation, alongside concurrent support for his mother, who had a psychiatric disorder. Adaptive behavior was assessed using the Japanese version of the Vineland Adaptive Behavior Scales, Second Edition (Vineland-II), focusing on the Socialization and Maladaptive Behavior domains. Although no marked improvement was observed at high school entry, socialization domain v-scores subsequently increased (e.g., coping skills from 6 to 15, interpersonal relationships from 9 to 14), and maladaptive behavior domain scores decreased (internalizing and externalizing both from 22 to 14) after graduation, in conjunction with employment transition support. Following continuous support spanning his school years through the prevocational period, he currently remains employed.

This case suggests that for students with neurodevelopmental disorders presenting with ER-SAC, changes in daily functioning that are not captured by short-term outcome measures can facilitate long-term competitive employment and social participation. In supporting these students, goals should extend beyond school reintegration. Continuous, uninterrupted support that anticipates future employment and social participation across transitional milestones, such as advancement to higher education and graduation, is essential.

## Introduction

In recent years, the increasing number of students with school absenteeism has become a major social concern in Japan, resulting in a growing need for support services [1]. School absenteeism is not merely a matter of being unable to attend school; it involves multifaceted difficulties, including disrupted daily life rhythms, reduced opportunities for social participation, and diminished self-esteem [2,3]. These challenges are particularly pronounced among students with neurodevelopmental disorders, such as autism spectrum disorder (ASD) and attention-deficit/hyperactivity disorder (ADHD), whose difficulties with interpersonal relationships and adaptation to their environment often contribute to prolonged absenteeism [4].

School absenteeism has traditionally been described using terms such as "school refusal," and its conceptualization has been discussed and refined over time [3]. School refusal behavior has been shown to be closely associated with emotional problems such as anxiety, depression, and stress [2]. Building on this understanding, the concept of emotion-related school attendance challenges (ER-SAC) has been proposed as a more inclusive, person-centered framework that conceptualizes attendance difficulties as rooted primarily in emotional factors, while encompassing behaviors previously classified as school refusal [5,6]. The present case, in which absenteeism was closely linked to anxiety and interpersonal tension against the background of neurodevelopmental disorders, is consistent with this conceptualization of ER-SAC.

The authors have provided continuous home-based occupational therapy (OT) support for students with school absenteeism presenting with ER-SAC [5,6]. Rather than aiming solely at a school return, this approach emphasizes comprehensive support, including the improvement of daily life rhythms, the enhancement of activities of daily living (ADLs), family support, academic assistance, and the promotion of social participation. For students with neurodevelopmental disorders, in addition to addressing anxiety and avoidance tendencies, the intervention focuses on building secure interpersonal relationships and fostering engagement that draws on the student’s interests. As a result, these efforts have facilitated school reengagement by enhancing self-efficacy and supporting the development of future-oriented goals.

However, most previous reports have focused on improving adaptation up to the point of returning to school or advancing to high school. In contrast, studies examining the long-term trajectories of these students, such as their post-graduation progress and transition to employment, remain limited. Particularly in contemporary Japan, where enrollment in correspondence high schools is rising and career paths are becoming more diverse, continuous support that extends beyond the school years through adolescence and young adulthood is critical. Nevertheless, few reports have specifically examined how home-based OT may contribute to reconstructing daily life among students with school absenteeism within a broader trajectory toward employment. This report describes the case of a student with ASD and ADHD who experienced ER-SAC and received continuous support centered on home-based OT. Through advancement to and graduation from high school, combined with the use of employment transition support, a disability welfare service in Japan, the student ultimately reached employment. Drawing on this case, we examine the long-term role of home-based OT in supporting students with school absenteeism, spanning from the school years through successful employment.

## Case presentation

The case involves a boy aged 12 years and 11 months who had been diagnosed with ASD and ADHD. He lived with his parents; however, his father was minimally involved in childcare, and the case had little interaction with him. During elementary and junior high school, he was enrolled in a special-needs class while attending regular classes for certain subjects, including Japanese and mathematics. He attended junior high school 2-3 days per week; however, his motivation to study was low, and he was absent for more than 30 days per year, meeting the criteria for school absenteeism.

He was introverted and had little interaction with anyone other than a few specific individuals. He experienced strong interpersonal tension and struggled with communication. At home, his daily routine centered entirely on online gaming; upon returning from school, he would immediately begin playing without changing his clothes. As a result, staying up late became habitual, disrupting his daily life rhythms and often leaving him unable to wake up in the morning or attend school. He also began neglecting his ADLs, often going to school without bathing or grooming. These changes in daily life rhythms and ADLs are summarized in Table 1.

**Table 1:** Changes in activities of daily living and daily functioning across intervention phases JHS: junior high school

Phase	Sleep–Wake Cycle	Bathing	Grooming	Academic/Vocational Engagement	Attendance
Phase I (JHS Grade 1)	Irregular	1–2 times/week	Poor	Minimal	Attended 2–3 days/week with frequent absences
Phase II (JHS Grade 2)	Improving	3–4 times/week	With prompting	Possible with support	Reduced absences
Phase III (JHS Grade 3)	Irregular	1–2 times/week	Poor	Declined	Not attending school
Phase IV (High School)	Improving	Almost daily	Increasing independence	Home-based learning	Minimal in-person attendance
Phase V (Vocational Transition)	Stable	Daily	Independent	Employment (gas station)	Attending five days/week

The primary caregiver, his mother, was undergoing treatment for a psychiatric disorder and demonstrated a tendency toward overprotective parenting. When reprimanded by his mother, he displayed a defiant attitude and, in an agitated state, would occasionally say, “I’ll kill myself.” However, once he regained his composure, he would apologize. This pattern suggested that these utterances were impulsive, situationally driven expressions of distress rather than reflections of genuine suicidal intent. His individual characteristics, the support process, and his relationship with his mother are summarized in Table 2.

**Table 2:** Intervention process for the case and his mother OTR: occupational therapist registered; ADLs: activities of daily living; OT: occupational therapy

Phase	Period	Key Characteristics and Challenges	Intervention Goals and Plan	Intervention and Case’s Response	Support for and Relationship with Mother
Phase 1	Jan–Mar, 1st Year of Junior High School	The case had low motivation to attend school and led a game-centered life at home. He was dependent on his mother, and his ADLs were neglected.	Goal: Establish a secure and trusting relationship with the case. Plan: Engage mainly through discussions about games, which interested him, while avoiding school-related topics.	The case did not show immediate resistance to the visits but was often found playing online games and voice-chatting with friends during the OTR’s visits. Engagement began by not rejecting this behavior and simply sharing the same space.	The OTR and the mother shared information regarding the case’s current situation and difficulties. Because the mother had a psychiatric disorder, her condition and her relationship with the father were also assessed.
Phase 2	Apr–Mar, 2nd Year of Junior High School	School attendance improved, but managing academic assignments and submissions remained difficult. Awareness of ADLs was also low, with bathing and grooming occurring only two to three times a week.	Goal: Maintain regular school attendance, complete academic assignments, and improve bathing and grooming habits. Plan: Without rejecting games, discuss assignment management and the importance of bathing and grooming, and positively acknowledge his attendance.	He became able to work on minimal assignments before examinations. His absences decreased, and he attended school regularly except when ill. He also became able to bathe and groom himself before going to school.	As the mother and the case had been sleeping in the same bed, the OTR proposed separating their bedrooms to promote his independence and ensure physical distance, to which the mother agreed.
Phase 3	Apr–Mar, 3rd Year of Junior High School	School absenteeism recurred following changes in the school environment. He had little concrete understanding of advancing to high school and did not feel the necessity of doing so.	Goal: Study toward high school advancement and select an appropriate school. Plan: Increase OT visits to twice a week and provide mental health and academic support; discuss the significance and necessity of advancing to high school.	He became able to engage in studying, focusing mainly on English, during the OTR’s visits. He also came to understand the necessity of advancing to high school.	As the mother’s psychological stress increased with the case’s renewed absenteeism, home-visit nursing for the mother was initiated.
Phase 4	1st–3rd Year of High School	He entered a correspondence high school but had difficulty completing academic assignments and managing submissions, with a low level of independence.	Goal: Engage proactively with high school assignments and graduate. Plan: Increase OT visits to three times a week to secure time for the case to work on assignments with the OTR; start with subjects he was good at, check submission deadlines, and create a study schedule together with the case.	In the first year, he could hardly work independently and required assistance from the OTR and his mother. In the second year, the number of subjects he could manage independently increased, and by the third year, he was able to submit assignments with minimal assistance. Additionally, bicycle-riding practice was introduced to secure opportunities to go out, expanding his range of activities, which enabled him to shop independently.	Mental health support for the mother through home-visiting nursing continued. The OTR exchanged information with the mother as appropriate regarding the case’s progress with assignments.
Phase 5	Employment Transition Support Services (2 years)	After graduating from high school, he began attending employment transition support. Although he interacted often with friends through game voice chat, his experience of group interpersonal interaction was limited, and his life skills as a member of society were underdeveloped.	Goal: Improve social skills and obtain employment. Plan: Provide continuous mental health support to facilitate daily attendance; discuss daily concerns and social norms, and positively reinforce his achievements.	He was initially anxious about attending in the morning but gradually became able to attend consistently. Although he sometimes felt discouraged by critical feedback from the employment transition support staff, he was able to consult the staff each time and regain his composure.	Mental health support for the mother through home-visiting nursing continued. The OTR monitored the case’s living situation through his mother as appropriate and encouraged her not to rush his job-seeking process.
Current status		Employed at a gas station and continues to work reliably.			

To assess his adaptive functioning, the Japanese version of the Vineland Adaptive Behavior Scales, Second Edition (Vineland-II) [7] was utilized. The Vineland-II is a standardized scale for measuring adaptive behavior and comprises four domains: Communication, Daily Living Skills, Socialization, and Motor Skills. The assessment is administered through a semi-structured interview with a guardian or other key informant. In addition to these four domains, an optional Maladaptive Behavior domain can be included to assess internalizing, externalizing, and socially deviant problems. In this report, we focused specifically on the Socialization and Maladaptive Behavior domains. The instrument used was the officially authorized Japanese version published by Nihon Bunka Kagaku Sha and procured through an authorized distributor in Japan. The Socialization domain comprises three subdomains, Interpersonal Relationships, Play and Leisure Time, and Coping Skills, all of which are directly relevant to the intervention goals in this case. Each item is rated on a three-point scale (2 = usually performs, 1 = sometimes or partially performs, 0 = never performs). In contrast, the Maladaptive Behavior domain evaluates the presence and frequency of problem behaviors, with higher scores indicating a greater frequency of maladaptive behavior. Subdomain results are expressed as v-scores (M = 15, SD = 3).

Phase I (January-March, first year of junior high school)

Home-based OT was provided once a week. Simultaneously, considering the mother’s psychological burden, weekly family support sessions were conducted by a separate occupational therapist registered (OTR) responsible for family support. Although the case did not display a rejecting attitude during the initial visit, he remained wary of the OTR. During visits, he typically continued playing online games and rarely responded to questions. To build rapport, the OTR engaged with him primarily through discussions of online gaming, directly leveraging his personal interests. During family support sessions, the OTR would listen to the mother’s daily concerns and discuss approaches for responding to her son’s behavioral challenges.

Phase II (April-March, second year of junior high school)

During home-based OT sessions, the OTR reviewed the case’s submission of academic assignments and discussed the importance of maintaining ADLs. Even when the case continued playing online games during the visits, the OTR did not discourage this behavior, focusing primarily on fostering a sense of security. Over this period, the case began working on his academic assignments when prompted, and his school absences gradually decreased. Although the mother experienced periods of psychological instability, she learned to maintain an appropriate distance from her son, which eased her overprotective tendencies.

Phase III (April-March, third year of junior high school)

At the start of the new school year, the case’s absenteeism recurred due to difficulties adapting to a new regular classroom environment and a change in his special-needs teacher. In June, a conference involving the special-needs class teacher, the mother, the OTR, and the case was held to discuss the feasibility of continued school attendance; however, securing stable attendance proved difficult. In response, the OTR initiated frequent discussions regarding high school advancement and proposed increasing home visits to twice a week to secure dedicated study time and foster social interaction. Although the case was initially reluctant to study, discussions about his future goals and significance of advancing to high school gradually made him more receptive. Consequently, home visits were increased to twice a week starting in September. Two OTRs shared responsibility for the intervention, dividing their roles into “mental health support” and “academic support.” Driven by this approach, the case progressively developed an interest in English, and by December, before his entrance examination, he had established a regular weekly study routine. Accordingly, his negative remarks about studying declined. With a new motivation to pursue high school education, he began visiting correspondence high schools (which require fewer in-person attendance days) in January and was ultimately admitted. Furthermore, to address the heightened parental stress accompanying the case’s renewed absenteeism, home-visiting nursing services for the mother were initiated during this period.

Phase IV (first to third year of high school)

After enrolling in a correspondence high school, the case experienced difficulty completing his academic assignments and managing submissions. Hence, home visits were increased to three times a week, with two sessions dedicated to “academic support” and one to “mental health support.” During his first year, completing assignments independently remained challenging, frequently requiring the assistance of the OTR and his mother. By the second year, he began managing a greater number of subjects on his own, allowing the OTR to intervene only with particularly difficult subjects. Consequently, he became increasingly proactive, taking ownership of his tasks while remaining mindful of submission deadlines. By the third year, he was able to complete assignments independently, keeping the OTR’s involvement to a minimum. In addition, to create opportunities to leave the house within his home-centered lifestyle, bicycle-riding practice was introduced. This successfully expanded his community mobility; for instance, he became able to shop on his own, reflecting a broadening of his activities, including instrumental activities of daily living.

Phase V (employment transition support, two years)

Following high school graduation, the case began attending employment transition support, a disability welfare service in Japan, to address his immature social skills and employment-related anxiety stemming from his limited school attendance at the correspondence high school. He initially attended the program three days a week; however, within two months, he was attending every weekday except holidays.

To facilitate a smooth transition, the OTR provided the employment transition support staff with detailed information regarding his daily routines and progress throughout junior high and high school. In addition, conferences involving the case, the OTR, and the support staff were held every six months to collaboratively set goals and review progress.

During this period, the case increasingly sought guidance from the OTR regarding appropriate communication and ways to demonstrate consideration for others. The OTR provided specific feedback on his interpersonal experiences and highlighted his improved social adaptation skills, framing this progress as mastery experiences.

Through the employment transition support program, he received continued training in social adaptation skills, including professional etiquette, grooming, and job-seeking strategies. Over time, these experiences enhanced his self-esteem and confidence regarding employment, leading to his current job at a gas station.

Currently, home-based OT continues once a week, with a primary focus on mental health support. In addition, the mother receives weekly home-visiting nursing services to assist in medication management and promote her understanding and acceptance of her son’s circumstances.

Over the course of the intervention, the case demonstrated improved Vineland-II Socialization scores and reduced scores in the Maladaptive Behavior domain (Table 3).

**Table 3:** Vineland adaptive behavior scales, second edition (Vineland-II) scores across the support period OT: Occupational therapy Note. Values are v-scale scores (M = 15, SD = 3). For the Socialization domain (Interpersonal Relationships, Play and Leisure Time, and Coping Skills), higher scores indicate better adaptive functioning. For the Maladaptive Behavior domain (Internalizing, Externalizing), lower scores indicate improvement.

Domain	Start of Home-Based OT	At High School Entry	After Employment
Interpersonal Relationships	9	9	14
Play and Leisure Time	10	8	15
Coping Skills	6	9	15
Internalizing	22	20	14
Externalizing	22	22	14

## Discussion

In this report, we described the long-term process by which a child with a neurodevelopmental disorder presenting with ER-SAC advanced to high school and ultimately reached employment, supported by continuous home-based OT within a multidisciplinary framework that included employment transition support. A distinctive feature of this case is that the intervention was not limited to a short-term response to ER-SAC but instead continuously addressed the individual’s long-term development trajectory, from the school years through the prevocational period.

During the early stages of intervention, the case was wary of the OTR and demonstrated minimal responsiveness, continuing to play online games during home visits. By using games, an activity of deep interest to him, as a medium of engagement and prioritizing rapport-building while avoiding rejecting interventions, a trusting relationship gradually developed. Students presenting with ER-SAC often exhibit avoidant behavior rooted in anxiety and interpersonal tension; therefore, ensuring psychological safety is important in the early stages of support [8]. In this case, an interest-mediated, non-rejecting approach is believed to have laid the groundwork for sustained, long-term support. From a theoretical standpoint, engaging the case through an activity of genuine personal interest can be understood as fostering a therapeutic alliance [8] and promoting occupational engagement, which together may have supported his emerging sense of autonomy and self-directed participation. This process is consistent with the emphasis, in self-determination theory, on autonomy and intrinsic motivation as drivers of sustained engagement [9].

Notably, the case's online gaming appeared to function not as an addictive behavior but as a means of social connection, a community in which he interacted with friends. Rather than restricting this activity, the OTR accepted it, and over the course of support, the case gradually developed the ability to regulate his own use, refraining from gaming during study time. This suggests that respecting a personally meaningful activity, rather than treating it as a problem to be eliminated, may support the development of self-regulation.

In addition to supporting the case directly, the continuous provision of support for his mother was equally vital. The mother was undergoing treatment for a psychiatric disorder and showed overprotective tendencies toward the case; through home-based OT and home-visiting nursing, efforts were made to reduce her psychological burden and establish an appropriate sense of distance between them. Given that the case's development of psychological independence lagged behind that of his peers, as reflected, for example, in his continuing to share a bed with his mother even after entering junior high school, the environmental adjustment of separating their bedrooms during his second year likely helped form a foundation that promoted his autonomy. This intervention reflects a family-centered approach, a hallmark of home-based OT, which facilitated psychological differentiation between the mother and child while fostering the case’s autonomy. Such direct intervention within the home environment is often difficult to achieve in clinic-based settings. In ER-SAC cases, the family environment has been reported to heavily influence a child’s emotional stability and behavioral development [10,11]. Consistent with these findings, family support appears to have been an indispensable component of the overall intervention strategy. Specifically, the occupational therapists and the visiting nurse shared information on the case's daily living situation and the mother's psychological state, enabling a coordinated approach in which the mother's stabilization and the case's daily routine and ADL improvement were supported in parallel. This division of roles-with occupational therapy addressing both the case's daily functioning and the mother's parenting concerns, and home-visiting nursing supporting the mother's mental health and medication management-illustrates a concrete mechanism through which multidisciplinary home-based support may reinforce a child's adaptive behavior.

Regarding the Vineland-II results, no marked improvement was observed in the Interpersonal Relationships or Play and Leisure Time subdomains at the time of high school entry. In fact, scores for Play and Leisure Time had declined since the start of the intervention. This decline may reflect the recurrence of ER-SAC during the third year of junior high school, as well as the shift to a home-centered learning environment after enrollment in the correspondence high school. As correspondence high schools require minimal attendance, opportunities to leave the home and interact with others tend to be naturally limited. Consequently, there may have been fewer real-world opportunities to practice adaptive behaviors related to socialization and leisure, which may explain the lack of improvement in these Vineland-II scores. This suggests that improvements in adaptive behavior depend not only on an individual’s abilities but also on actual opportunities for interpersonal engagement and participation in activities. When interpreting these v-scores, it should be noted that a change of approximately three points (one standard deviation) is generally regarded as clinically meaningful in group-based research [5,7]. In this case, however, even smaller score changes may reflect meaningful shifts in daily adaptive functioning, given the sensitivity of v-scores to real-life behavioral changes and the single-case nature of this report [5]. Accordingly, the score changes observed here should be interpreted in conjunction with the qualitative behavioral changes documented across the intervention phases, rather than on the basis of numerical values alone.

However, the plateau in adaptive behavior during this period does not imply that the support was ineffective; rather, it reflects an intentional approach based on phase-by-phase goal setting. In this instance, the period of enrollment in a correspondence high school was not intended to accelerate the acquisition of social skills; instead, the primary goals were high school graduation and the independent completion of academic assignments. The full-fledged development of social skills, including interpersonal interaction, was deliberately deferred to the subsequent employment transition support phase, a strategic postponement of social skill acquisition. Meanwhile, although opportunities for activity were limited within his home-centered lifestyle, bicycle riding was introduced to facilitate greater engagement in out-of-home activities. From an OT perspective, incorporating bicycle riding was not merely physical training but the strategic enablement of a meaningful occupation. It modified the person-environment-occupation fit, allowing him to transition from a home-centered lifestyle to active community mobility. This intervention yielded improvements in real-life functional abilities, such as the ability to shop independently. The selective incorporation of feasible occupations within a constrained environment may have provided a bridge from his home-centered lifestyle toward employment transition, forming the foundation for the marked improvement in coping skills (from 6 to 15) in the subsequent Phase V.

From the high school period through the employment transition support phase, scores in the Socialization domain, including coping skills, improved, while scores for internalizing and externalizing problems decreased. As shown in Table 1, ADLs such as daily life rhythms, bathing, and grooming also improved gradually. During the employment transition support period, he was able to attend the program five days a week. Although his daily functioning had temporarily declined following the recurrence of ER-SAC during his third year of junior high school, the subsequent progress from high school onward suggests that daily functioning in children with neurodevelopmental disorders presenting with ER-SAC is highly susceptible to changes in environmental conditions and psychological states.

During the shift to employment transition support, the OTR deliberately adopted a policy of minimizing environmental accommodations. This approach was based on the view that excessive reliance on structured support could hinder adaptation to a competitive employment environment. Accordingly, competitive employment rather than disability-supported employment was envisioned as the long-term goal from the outset. However, this did not mean that support was withdrawn. Prior to the transition, the OTR provided the employment transition support staff with detailed information regarding the case’s living situation and progress during his school years. In addition, goals were continuously established, and progress was reviewed through conferences held every six months. Within this supportive environment, the case increasingly sought guidance from the OTR to refine his manner of speaking and evaluate how he demonstrated consideration for others. This behavior likely reflects significant improvements in self-awareness and social self-understanding during interpersonal interactions. Children with neurodevelopmental disorders presenting with ER-SAC often experience anxiety regarding their relationships with others, yet they rarely have opportunities to objectively reflect on their own interpersonal behavior [12,13]. In this instance, the OTR provided the case with explicit feedback regarding his interpersonal growth, framing these interactions as mastery experiences to bolster his self-efficacy. Overall, the support provided through information sharing and regular goal setting while minimizing environmental accommodations, the practical social experiences gained through employment transition support, and the OTR’s continuous feedback are believed to have contributed to the improvement of the case’s proactive social adaptation skills.

Most previous reports on interventions for school absenteeism have treated improvement in adaptation up to school return or educational advancement as the primary outcome. In contrast, this study is significant because it presents a support process extending beyond high school graduation to employment, an outcome that has rarely been reported in the literature. In this case, although no substantial changes in Vineland-II scores were observed at the time of high school entry, the case ultimately reached employment over the long term. This finding suggests that changes in daily functioning not captured by short-term numerical measures may contribute to long-term social participation.

Several limitations should be considered when interpreting the findings of this study. First, as this is a single-case report without a control group, caution must be exercised when generalizing the results. Second, because home-based OT, home-visiting nursing, and employment transition support were provided concurrently, the observed changes cannot be attributed solely to home-based OT. Third, since the Vineland-II relies primarily on a semi-structured interview with a guardian as the main informant, the potential influence of the informant’s subjectivity cannot be entirely ruled out. Fourth, the case received support from facilities operated by a single corporation in an urban area in Japan. Consequently, these findings may reflect localized support structures, and the generalizability of this model to rural regions or to institutions with different organizational cultures remains to be determined. Future research should therefore examine similar support processes across multiple cases and systematically evaluate long-term outcomes. At the same time, the concurrent provision of home-based OT, home-visiting nursing, and employment transition support should be understood not merely as a methodological limitation but also as a clinical reality: supporting a child with ER-SAC toward long-term social participation may inherently require sustained, multidisciplinary collaboration across educational, welfare, and healthcare domains. Future practice and research may therefore focus on optimizing such collaborative frameworks rather than isolating the effect of a single intervention.

## Conclusions

This case illustrates a long-term trajectory in which continuous support centered on home-based OT, provided within a multidisciplinary framework, may have facilitated the process by which a child with a neurodevelopmental disorder presenting with ER-SAC graduated from high school and ultimately reached employment. Although no marked improvements were observed in standardized measures at the time of high school entry, the eventual attainment of employment suggests that the accumulation of gains in daily functioning, which may not be reflected in short-term outcome measures alone, can contribute to long-term social participation. In supporting children with neurodevelopmental disorders presenting with ER-SAC, it is important not to regard school return as the sole objective. Rather, support should be guided by a long-term perspective that anticipates future employment and social participation, with collaboration among relevant institutions to ensure continuity of support during key transitional milestones, such as educational advancement and graduation. Ultimately, this case underscores that sustained occupational engagement and continuous support across developmental transitions can play a meaningful role in helping adolescents with neurodevelopmental disorders move toward social participation and adult roles.

## References

[1] (2026). Survey results on various student guidance issues, including problem behaviors and school absenteeism among children and students in Fy2024 (Document in Japanese). https://www.mext.go.jp/content/20260116-mxt_jidou02-100002753_1_3.pdf.

[2] Gonzálvez C, Kearney CA, Jiménez-Ayala CE, Sanmartín R, Vicent M, Inglés CJ, García-Fernández JM (2018). Functional profiles of school refusal behavior and their relationship with depression, anxiety, and stress. Psychiatry Res.

[3] Elliott JG, Place M (2019). Practitioner review: School refusal: developments in conceptualisation and treatment since 2000. J Child Psychol Psychiatry.

[4] Munkhaugen EK, Torske T, Gjevik E, Nærland T, Pripp AH, Diseth TH (2019). Individual characteristics of students with autism spectrum disorders and school refusal behavior. Autism.

[5] Oto F, Matsuda N, Ito K, Morikawa A, Fujii H (2025). Home-based occupational therapy for emotion-related school attendance challenges: two case studies. Cureus.

[6] Oto F, Matsuda N, Ito K, Ito S, Morikawa A, Fujii H (2025). Effectiveness of visiting occupational therapy for a junior high school student refusing to attend school due to internet game addiction. Asian J Occup Ther.

[7] Sparrow SS, Cicchetti DV, Balla DA (2005). Vineland Adaptive Behavior Scales, Second Edition (Vineland-II). Circle Pines, MN: American Guidance Service.

[8] Klebanoff SM, Rosenau KA, Wood JJ (2019). The therapeutic alliance in cognitive-behavioral therapy for school-aged children with autism and clinical anxiety. Autism.

[9] Ryan RM, Deci EL (2000). Self-determination theory and the facilitation of intrinsic motivation, social development, and well-being. Am Psychol.

[10] Chockalingam M, Skinner K, Melvin G, Yap MB (2023). Modifiable parent factors associated with child and adolescent school refusal: a systematic review. Child Psychiatry Hum Dev.

[11] Cronshaw G, Midouhas E (2024). Harsh parenting and trajectories of emotional and behavioural difficulties in autistic children. J Autism Dev Disord.

[12] Bölte S (2025). Social cognition in autism and ADHD. Neurosci Biobehav Rev.

[13] Miranda A, Berenguer C, Roselló B, Baixauli I, Colomer C (2017). Social cognition in children with high-functioning autism spectrum disorder and attention-deficit/hyperactivity disorder. Associations with executive functions. Front Psychol.

